# The long noncoding RNA PCGEM1 promotes cell proliferation, migration and invasion via targeting the miR-182/FBXW11 axis in cervical cancer

**DOI:** 10.1186/s12935-019-1030-8

**Published:** 2019-11-20

**Authors:** Qian Zhang, Jindan Zheng, Lili Liu

**Affiliations:** Gynecology Department, The First Affiliated Hospital, Jinzhou Medical University, No.2 of the People Street, Guta District, Jinzhou, 121001 Liaoning China

**Keywords:** ceRNA, FBXW11, lncRNA, miR-182

## Abstract

**Background:**

Cervical cancer (CC) is the fourth leading cause of cancer-associated death in women worldwide. Recently, long noncoding RNA (lncRNA) prostate cancer gene expression marker 1 (PCGEM1) has been demonstrated to involve in the initiation and progression of human cancers. However, to date, the clinical and functional significance of PCGEM1 expression in CC progression remains unknown.

**Methods:**

qRT-PCR was performed to investigate PCGEM1 expression levels in CC tissues and cell lines. The effect of PCGEM1 on CC cells was assessed by gain- and loss-of-function assays. MS2-binding sequences-MS2-binding protein-based RIP assay (MS2-RIP), RNA pull-down and Luciferase reporter assays were performed to investigate the interaction between PCGEM1 and miR-182. The association between miR-182 and F-box and WD repeat domain containing 11 (FBXW11) was verified by luciferase reporter assay. The effect of PCGEM1 on the NF-κB and β-catenin/TCF signaling pathways was determined by luciferase reporter assay.

**Results:**

Our present study showed that PCGEM1 was significantly upregulated in CC tissues and cell lines. Overexpression of PCGEM1 was correlated with advanced International Federation of Gynecology and Obstetrics (FIGO) stage, lymph node, distant metastasis and poor prognosis in CC patients. Functionally, PCGEM1 promoted cell proliferation, cell cycle progression, migration and invasion, while suppressed cell apoptosis in CC cells. Further mechanistic investigation revealed that PCGEM1 associated with miR-182 and suppressed its expression. PCGEM1 could act as a competing endogenous (ceRNA) of oncogene F-box and WD repeat domain containing 11 (FBXW11) for miR-182 in CC cells. Additionally, PCGEM1 was capable to activate the NF-κB and β-catenin/TCF signaling pathways, which was reversed by inhibition of FBXW11.

**Conclusion:**

In conclusion, our findings demonstrated that PCGEM1-miR-182-FBXW11 axis play an important role in CC progression, and indicated a promising therapeutic target for CC patients.

## Background

Cervical cancer (CC) is the fourth leading cause of cancer-associated death in women worldwide [1]. Approximately 530,000 new cases are diagnosed with CC every year, and 85% of the deaths occur in underdeveloped or developing countries [2]. Most early CC could be cured by surgical resection. However, for those CC patients in advanced stages, there are no effective therapeutic strategies [3]. Hence, further investigation are needed to reveal the molecular mechanism responsible for CC initiation and progression, which may be greatly helpful for identifying effective therapeutic approaches for CC patients.

Long noncoding RNAs (lncRNAs) are a heterogeneous class of transcripts larger than 200 nucleotides without protein-coding ability [4]. LncRNAs play crucial roles in controlling gene expression involving several cellular processes of human cancers, such as proliferation, migration, invasion, drug resistance, autophagy and angiogenesis [5, 6]. The mechanisms of lncRNAs include gene regulation *in cis* or *in trans*, and regulation of their interacting proteins [7–9]. Previous studies have provided evidence suggesting that the deregulation of lncRNAs participate in the initiation and progression of CC, including that of GAS5, CRNDE, SPRY-IT1 and CCAT1 [10–13]. Recently, lncRNA prostate cancer gene expression marker 1 (PCGEM1) has been identified as an oncogenic gene in human cancers. PCGEM1 was first found to be highly expressed in prostate cancer and promotes cell proliferation [14, 15]. PCGRM1 exerts oncogenic effects in prostate cancer cells through acting as a competing endogenous RNA (ceRNA) for some microRNAs, such miR-145 and miR-148a [16, 17]. Besides, PCGEM1 expression level is overexpressed in epithelial ovarian cancer tissues. PCGEM1 enhances ovarian cancer cell proliferation, migration, and invasion, but decreased cell apoptosis through upregulating RhoA, YAP, MMP2, Bcl-xL, and P70S6K expression [18]. In endometrial carcinoma, PCGEM1 upregulates STAT3 expression by acting as a ceRNA for miR-129 [19]. Moreover, PCGEM1 is capable to induce epithelial–mesenchymal transition (EMT) and metastasis via increasing SNAI1 expression in gastric cancer cells [20]. However, it is unclear whether PCGEM1 exerts similar function in CC tumorigenesis and development.

In present study, we first reported that lncRNA PCGEM1 was upregulated in CC tissues and cells, which may serve as a potential prognostic indicator for CC patients. We further explored the effects of PCGEM1 on the phenotypes of CC cells. Moreover, mechanistic investigation revealed that PCGEM1 could act as a ceRNA to regulate oncogene F-box and WD repeat domain containing 11 (FBXW11) expression by sponging miR-182 in CC cells. Taken together, our study provides the first evidence of the existence of a PCGEM1-miR-182-FBXW11 axis, which may be utilized as a promising therapeutic target for CC.

## Material and method

### Clinical specimens

Sixty-eight fresh CC tissues and their adjacent normal cervical tissues were obtained from patients diagnosed with cervical cancer in The First Affiliated Hospital of Jinzhou Medical University. All the tissue specimens were stored at − 80 °C until use. RNA later solution (Invitrogen™) was used to avoid the degradation of RNA, and all of the tissues were detect in a short time after resection from patients. This study was conducted with the approval of the Ethics committee of The First Affiliated Hospital of Jinzhou Medical University. The research has been carried out in accordance with the World Medical Association Declaration of Helsinki. Informed consent was obtained from all patients.

### Cell culture

A normal human cervix epithelial cell line (Ect1/E6E7) and four cervical cancer cell lines (C33A, HeLa, SiHa, and CaSki) were purchased from American Type Culture Collection (Manassas, USA). The STR profiling and mycoplasma testing in all cervical cancer cell line was checked. Cells were routinely cultured in Dulbecco’s Modified Eagle Medium (DMEM) (Gibco, USA) supplemented with 10% fetal bovine serum, 100 U/mL penicillin, and 100 µg/mL streptomycin in a humidified atmosphere of 5% CO_2_ at 37 °C.

### Transfection

siRNAs targeting PCGEM1, FBXW11 and a negative control (siNC) were purchased from Ribobio Company (Guangzhou, China). miR-NC (control), miR-182 mimics, miR-182 inhibitors (inh-182) were also obtained from GenePharma Technology (Shanghai, China). PCGEM1 overexpression plasmids (pcDNA3.1-PCGEM1) were purchased from Genearray Biotechnology (Shanghai, China). Transfections were performed using lipofectamine 2000 (Invitrogen, USA) according to the manufacturer’s instructions. After 48 h, the cells were used for further experiments.

### Western blot analysis

Cells were lysed using RIPA buffer (Beyotime, Beijing, China). A total of 20 μg protein samples were separated using SDS-PAGE, and then transfer onto PVDF membranes (Millipore). The membranes were blocked by 5% non-fat milk, and incubated with antibodies: anti-FBXW11 (Abcam), or anti-GAPDH (Proteintech), at 4 °C overnight. After wash, the membrane was probed with matched secondary antibody, and then the proteins were visualized using Immobilon Western Chemiluminescent HRP substrate (Millipore).

### RNA extraction and quantitative real-time PCR (qRT-PCR)

Total RNA from CC tissues and cells was isolated by using Trizol reagent (Invitrogen, CA). The cDNA was synthesized from total RNA using the cDNA Reverse Transcription Kit (Takara). qRT-qPCR was performed using a StepOne Real-Time PCR System (Applied Biosystems, USA). Relative expression was normalized to GAPDH and was calculated by 2^−ΔΔCt^ method. The primer sequences were shown as follow: GAPDH (forward: GGTGTGAACCATGAGAAGTATGA, reverse: GAGTCCTTCCACGATACCAAAG), PCGEM1 (forward: CTGTGTCTGCAACTTCCTCTAA, reverse: TCCCAGTGCATCTCGTAGTA), cyclinD1 (forward: CCTCTCCCATGACCACAATATC, reverse: GAGAATCCCAAAGGACCAGAC), IL-6 (forward: GGAGACTTGCCTGGTGAAA, reverse: CTGGCTTGTTCCTCACTACTC), MMP9 (forward: GAACTTTGACAGCGACAAGAAG, reverse: CGGCACTGAGGAATGATCTAA), CD44 (forward: AATGGTCGCTACAGCATCTC, reverse: GCAAACTGCAGGTCTCAAATC), Bcl-xL (forward: GGTGGTTGACTTTCTCTCCTAC, reverse: TCTCCGATTCAGTCCCTTCT), myc (forward: GCTGCTTAGACGCTGGATTT, reverse: GAGTCGTAGTCGAGGTCATAGTT), MMP7 (forward: CACTGTTCCTCCACTCCATTTA, reverse: GACATCTACCCACTGCAAGTATAG), Axin-2 (forward: CTTATCGTGTGGGCAGTAAGA, reverse: GTTCTCGGGAAATGAGGTAGAG), TCF-1 (forward: ACCTATGACTCCGTCGATCTAT, reverse: TCAGCCCATCTCTGACCTAT).

### Cell proliferation assay

3 × 10^3^ cells were seeded into 96-well plates. At indicated time point, cell proliferation was measured using the Cell Counting Kit-8 reagent (CCK-8, Dojindo, Kyushu, Japan). Briefly, 10 μL CCK-8 reagent was added into each well of cell culture, and the plates were further incubated for another 2 h. The absorbance at 450 nm was evaluated by a microplate reader. For colony formation assay, 2 × 10^3^ cells were plated in six-well plates for 2 weeks. Cells were fixed in 4% paraformaldehyde for 10 min and then stained by 0.1% crystal violet for 30 min. Experiments were repeated at least three times.

### Cell apoptosis detection

Apoptotic cells were evaluated by ANXA5 and propidium iodide (PI) staining (Invitrogen, A13201) according to the manufacturer’s instructions, and analyzed by flow cytometry (Beckman Coulter, USA).

### Cell cycle detection

Cells were trypsinized and resuspended, fixed overnight at 4 °C in 70% ethanol, stained with propidium iodide, and then analyzed by flow cytometry (Beckman Coulter, USA).

### Cell migration and invasion assays

To detect cell migration and invasion, the cells were pretreated with mitomycin C (10 μg/mL) for 1 h to eliminate the influence of cell proliferation. The transwell chambers with 8-μm pores were obtained from Corning (Corning, NY). For cell migration detection, the transfected cells were harvested and resuspended in 100 μL serum-free medium and then transferred to the upper chambers. 500 μL DMEM supplemented with 10% FBS was added to the lower chamber. After incubation for 24 h, the Transwell membrane was fixed with 4% paraformaldehyde, stained with 0.1% crystal violet for 30 min, and then counted under a light microscope. For the invasion assay, the Transwell membrane (filter) was precoated with Matrigel BD Biosciences) at 37 °C overnight; the remaining experimental procedures were similar to the migration assay.

### RNA immunoprecipitation (RIP) and MS2-binding sequences-MS2-binding protein-based RIP assay (MS2-RIP)

For RIP assay, cells were transfected with miR-182 or miR-NC. After 48 h, cells were used to perform RIP experiments by using an AGO2 antibody and the Magna RIP™ RNA-Binding Protein Immunoprecipitation Kit according to the according to the manufacturer’s instructions.

MS2-RIP assay was performed as previously described [21]. Cells were transfected with pcDNA3.1-MS2, pcDNA3.1-PCGEM1-MS2, or pcDNA3.1-PCGEM1-mut-MS2 (mutation in miR-182 binding sites). After 48 h, cells were used to perform RIP by using an anti-GFP antibody (Abcam) and the Magna RIP RNA-Binding Protein Immunoprecipitation Kit (Millipore) according to the manufacturer’s instructions. Then the co-precipitated RNAs level was analyzed by qRT-PCR.

### RNA pull-down assay

RNA pull-down assay was performed as previously described [22]. In brief, PCGEM1 or PCGEM1-mut were in vitro transcribed, respectively, and biotin-labeled with the Biotin RNA Labeling Mix (Roche) and T7 RNA polymerase (Roche), treated with RNase-free DNase I (Roche), and purified with an RNeasy Mini Kit (Qiagen, Valencia, CA). 1 mg of whole-cell lysates were incubated with 3 μg of purified biotinylated transcripts for 1 h at 25 °C, then the complexes were isolated with streptavidin agarose beads (Invitrogen). The RNA present in the pull-down material was detected by qRT-PCR analysis.

### Luciferase reporter assays

The wild-type or mutant PCGEM1 was cloned into pmirGLO plasmid (named pmirGLO-PCGEM1 and pmirGLO-PCGEM1-mut). Cells were co-transfected with pmirGLO-PCGEM1 or pmirGLO-PCGEM1-mut and miR-NC or miR-182. After 48 h, Luciferase activities were measured using the Dual-Luciferase Reporter Assay System (Promega, USA). To detect the effect of PCGEM1 on FBXW11 3′UTR, the FBXW11 3′UTR was cloned into pmirGLO plasmid (named pmirGLO-FBXW11). Cells were co-transfected with pmirGLO-FBXW11 and miR-NC or miR-182. After 48 h, Luciferase activities were measured using the Dual-Luciferase Reporter Assay System (Promega, USA). NF-κB and β-catenin/TCF firefly luciferase reporter construct (TOPflash) and pRL-TK reporter plasmid encoding Renilla luciferase was purchased from Promega. Cells were plated in a 24-well plate and cotransfected with PCGEM1, NF-κB or β-catenin/TCF firefly luciferase reporter, and pRL-TK (Promega) by using Lipofectamine 2000. The pRL-TK vector was used as an internal control. 48 h later, cells were collected and analyzed using the Dual-Luciferase Reporter Assay System (Promega).

### Statistical analysis

All experiments were performed at least three times. Data are presented as the mean ± standard error of mean (SEM). Statistical analysis was performed by using the SPSS 23.0 software. Student’s t test (for two groups comparison) or one-way analysis of variance (ANOVA; for more than two groups comparison) was utilized to analyze significant difference. The χ^2^ test was carried out to explore the correlation between PCGEM1 levels and clinical features. Kaplan–Meier method and log-rank test was used to assess overall survival. p < 0.05 was considered statistically significant.

## Result

### PCGEM1 is highly expressed in CC tissues and predicts poor prognosis

**Fig. 1:**
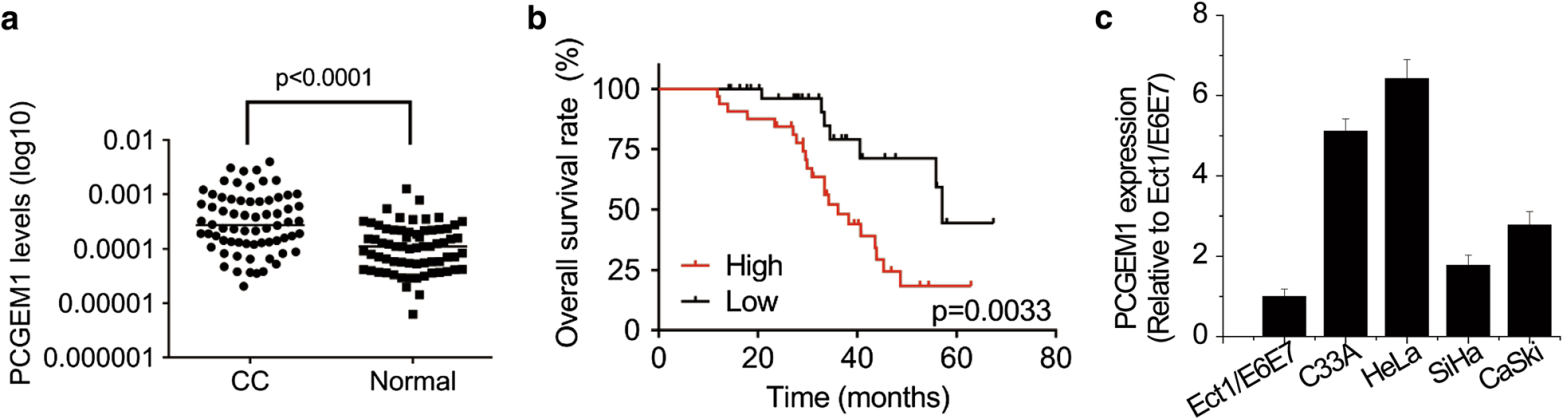
PCGEM1 is highly expressed in CC tissues and predicts poor prognosis. **a** The expression of PCGEM1 in 68 pairs of CC and normal cervical tissues was determined by qRT-PCR. **b** Kaplan–Meier survival curves and log-rank tests were used to assess the relationship between PCGEM1 levels and overall survival time of CC patients. The median of PCGEM1 expression levels in CC tissues was taken as cutoff. **c** PCGEM1 levels in four CC cells, including C33A, HeLa, SiHa, and CaSki, and Ect1/E6E7 cells (normal human cervix epithelial cells) were analyzed by qRT-PCR

To verify the expression levels of PCGEM1 in human CC tissues, the expression of PCGEM1 in 68 pairs of CC and normal cervical tissues was determined by qRT-PCR. As shown in Fig. 1a, PCGEM1 expressions were significantly increased in CC tissues compared with normal tissues. In addition, the association between PCGEM1 expression and clinicopathological features of CC patients was analyzed. It was found that the elevated PCGEM1 expression was correlated with advanced International Federation of Gynecology and Obstetrics (FIGO) stage, lymph node and distant metastasis (Table 1). Kaplan–Meier survival curves showed that patients with high PCGEM1 levels presented with worse overall survival than patients with low PCGEM1 levels (Fig. 1b). Moreover, we detected the PCGEM1 levels in four CC cells, including C33A, HeLa, SiHa, and CaSki, and Ect1/E6E7 cells (normal human cervix epithelial cells). The results demonstrated a higher expression of PCGEM1 in these four CC cells, when compared with the Ect1/E6E7 cells (Fig. 1c). These data showed that the expression of PCGEM1 was upregulated in CC tissues and cell lines, indicating PCGEM1 might be involved in the CC progression.

**Table 1:** The correlation between PCGEM1 expression and clinicopathological parameters in cervical cancer patients

Clinical parameters	PCGEM1		p value
	High	Low	
Age (years)			
≤ 40	15	19	0.332
> 40	19	15	
Size (cm)			
≥ 4	18	15	0.467
< 4	16	19	
Differentiation			
Well/moderate	15	18	0.467
Poor	19	16	
FIGO stages			
I–II	11	25	0.001
III–IV	23	9	
Lymphatic metastasis			
Yes	22	12	0.009
No	12	24	
Distant metastasis			
Yes	24	15	0.027
No	10	19	
Papillomavirus (HPV)			
Yes	20	17	0.465
No	14	17	

### PCGEM1 promotes CC cell proliferation, inhibits cell apoptosis and induces cell cycle progression

**Fig. 2:**
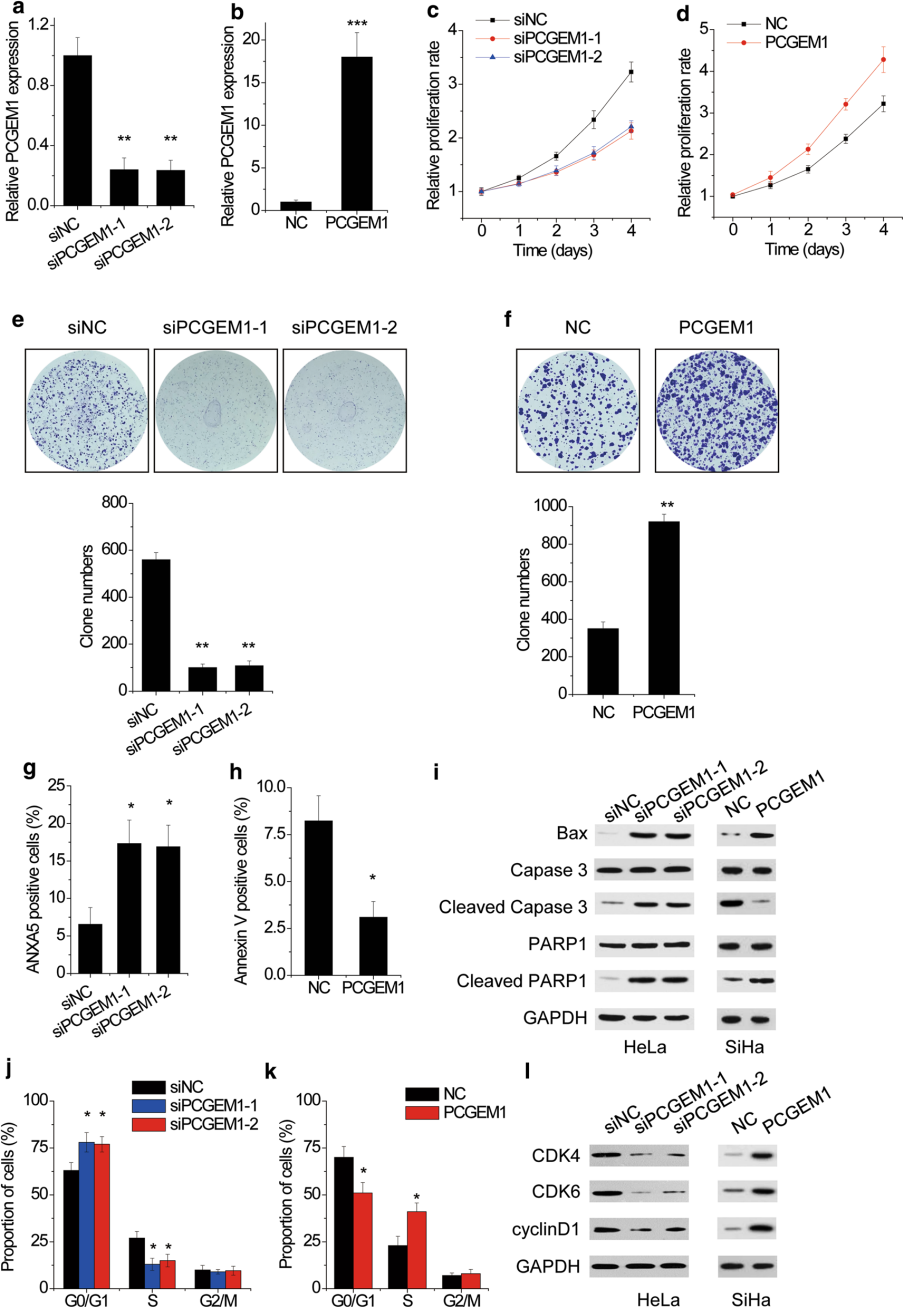
PCGEM1 promotes CC proliferation. **a** HeLa cells who had highest PCGEM1 were transfected with negative control siRNA (siNC) or two different specific siRNAs targeting PCGEM1 (siPCGEM1-1 and siPCGEM1-2). After 48 h, the PCGEM1 levels were determined by qRT-PCR. **b** SiHa cells who had lowest expression level of PCGEM1 were transfected with pcDNA3.1 (NC) or pcDNA3.1-PCGEM1 (PCGEM1) After 48 h, the PCGEM1 levels were determined by qRT-PCR. **c** The proliferation of HeLa cells with or without PCGEM1 knockdown was assessed by CCK-8 assay. **d** The proliferation of SiHa cells with or without PCGEM1 overexpression was assessed by CCK-8 assay. e The capability of clone formation is decreased in the siPCGEM1 group. **f** The capability of clone formation is increased in the PCGEM1 group. **g** ANXA5 and PI staining showed increased apoptosis of HeLa cells when PCGEM1 was silenced. **h** ANXA5 and PI staining showed decreased apoptosis of SiHa cells when PCGEM1 was overexpressed. **i** Western blot analysis showing the expression levels of caspase-3, PARP, and Bax following PCGEM1 alteration in HeLa and SiHa cells, respectively. **j** Flow cytometry analysis demonstrated that HeLa cells in S-phase population were significantly decreased when PCGEM1 was silenced. **k** Flow cytometry analysis demonstrated that SiHa cells in S-phase population were significantly increased when PCGEM1 was overexpressed. **l** Western blot analysis showed the expression levels of CDK4, CDK6, and cyclinD1 following PCGEM1 alteration in HeLa and SiHa cells, respectively. *p < 0.05, **p < 0.01, ***p < 0.001

To evaluate the roles of PCGEM1 in CC, overexpression and knockdown experiments were conducted. HeLa cells who had highest PCGEM1 were transfected with two different specific siRNAs (Fig. 2a), and SiHa cells who had lowest expression level of PCGEM1 were transfected with functional pcDNA3.1-PCGEM1 (Fig. 2b). The CCK-8 assay showed that knockdown of PCGEM1 significantly inhibited the proliferation of HeLa cells (Fig. 2c), while overexpression of PCGEM1 increased the proliferation of SiHa cells (Fig. 2d). A colony formation assay also confirmed that PCGEM1 knockdown markedly suppressed the colony formation ability in HeLa cells (Fig. 2e), whereas ectopic expression of PCGEM1 increased the colony formation number in SiHa cells (Fig. 2f).

We then detected apoptosis by flow cytometry analysis in CC cells. The results showed that deletion of endogenous PCGEM1 expression significantly promoted apoptosis in HeLa cells (Fig. 2g), while overexpression of PCGEM1 inhibited apoptosis in SiHa cells (Fig. 2h). Consistent with the flow cytometry data, the protein levels of Bax, cleaved PARP1, and cleaved caspase 3, the apoptosis protein markers, increased in PCGEM1 knockdown HeLa cells, but decreased in PCGEM1 overexpressing SiHa cells (Fig. 2i).

To further gain insights into the mechanism by which PCGEM1 promotes CC cell proliferation, flow cytometry was used to analyze differences in cell cycle distributions following PCGEM1 alteration. It was found that PCGEM1 knockdown significantly induced G1/S arrest in HeLa cells (Fig. 2j), while PCGEM1 overexpression drove progression beyond the G1/S transition in SiHa cells (Fig. 2k). Consistent with the flow cytometry data, the expression of G1/S phase checkpoint protein, such as CDK4, CDK6, and cyclinD1, was significantly markedly downregulated in HeLa cells with PCGEM1 knockdown and increased when PCGEM1 was overexpressed (Fig. 2l). Together, these data strongly demonstrated that PCGEM1 promotes cell proliferation by facilitating cell cycle progression and inhibiting cell apoptosis in CC.

### PCGEM1 enhances CC cell migration and invasion, and induces EMT

**Fig. 3:**
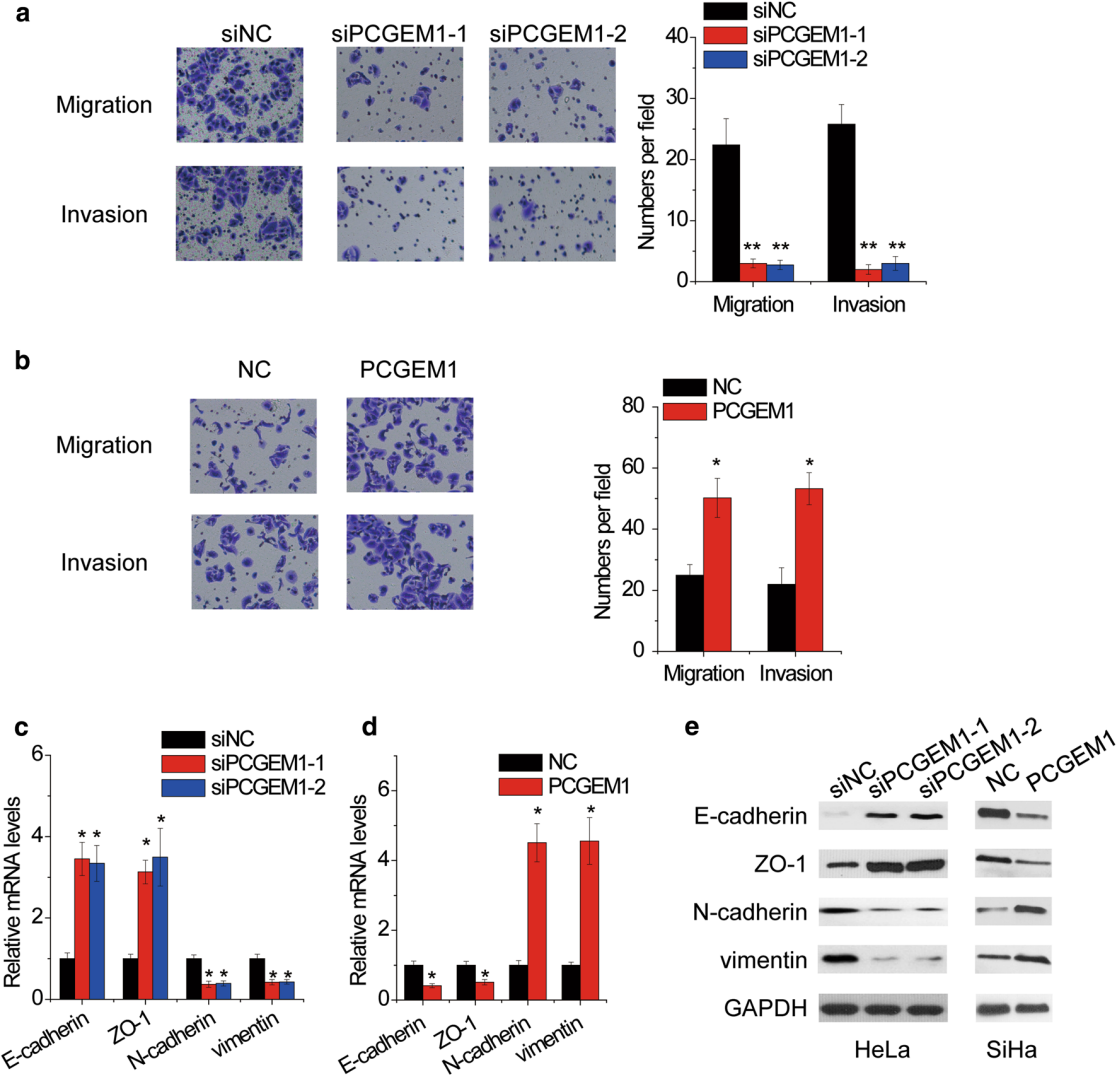
PCGEM1 enhances CC cell migration and invasion, and induces EMT. **a** Transwell assay was performed in HeLa cells transfected with two different siRNAs targeting PCGEM1. The number of cells that migrated or invaded was counted in ten different fields. **b** Transwell assay was performed in SiHa cells transfected with PCGEM1. The number of cells that migrated or invaded was counted in ten different fields. **c** qRT-PCR analysis of the epithelial (E-cadherin and ZO-1) and mesenchymal markers (N-cadherin and vimentin) in HeLa cells transfected with control or PCGEM1 siRNAs. **d** qRT-PCR analysis of the epithelial (E-cadherin and ZO-1) and mesenchymal markers (N-cadherin and vimentin) in SiHa cells transfected with empty vector or PCGEM1. **e** Western blot analysis of the epithelial (E-cadherin and ZO-1) and mesenchymal markers (N-cadherin and vimentin) in HeLa and SiHa cells with PCGEM1 alteration, respectively. *p < 0.05, **p < 0.01

A transwell assay demonstrated that knockdown of PCGEM1 significantly suppressed cell migration and invasion in HeLa cells (Fig. 3a), while ectopic expression of PCGEM1 markedly facilitated cell migration and invasion in SiHa cells (Fig. 3b). Since EMT progression is important for migration and invasion of CC cells, we tested whether PCGEM1 could induce EMT in CC cells. The qRT-PCR and western blotting analysis demonstrated that knockdown of PCGEM1 significantly reduced the mRNA and protein expression of mesenchymal markers (N-cadherin and vimentin), but increased the expression of epithelial markers (E-cadherin and ZO-1) in HeLa cells (Fig. 3c, e). In contrast, ectopic expression of PCGEM1 reduced the expression of epithelial marker E-cadherin and ZO-1, while increased mesenchymal marker N-cadherin and vimentin compared with control group in SiHa cells (Fig. 3d, e). Taken together, these results suggested that PCGEM1 facilitated CC cell migration and invasion.

### PCGEM1 directly interacts with miR-182

**Fig. 4:**
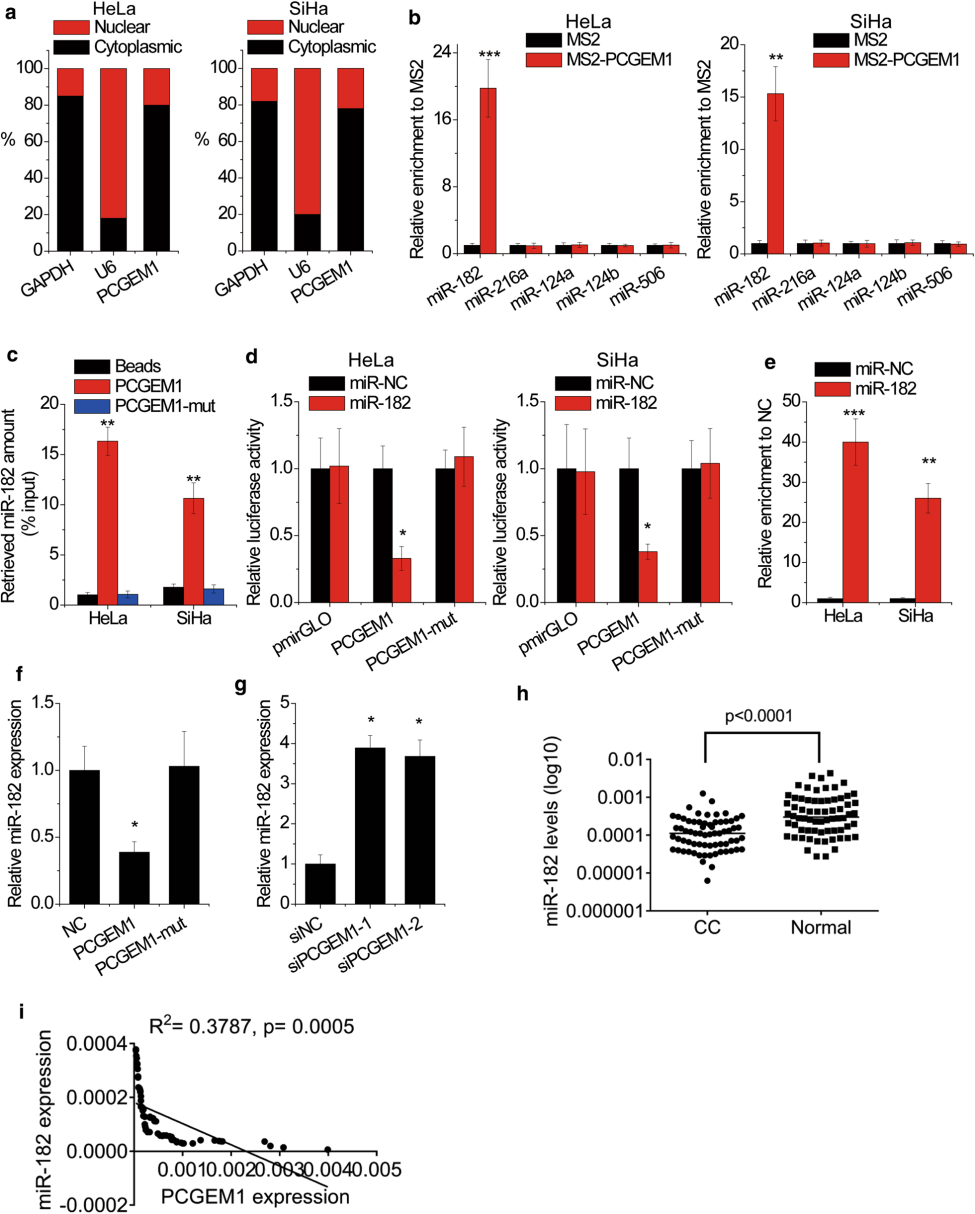
PCGEM1 directly interacts with miR-182. **a** Cytoplasmic and nuclear RNA fraction assay showed the cellular location of PCGEM1 in CC cells. U6 RNA served as a positive control for nuclear gene expression. GAPDH mRNA served as a positive control for cytoplasmic gene expression. **b** MS2-RIP followed by qRT-PCR was conducted to detect endogenous microRNAs associated with lncRNA PCGEM1. **c** HeLa and SiHa cell lysates were incubated with biotin-labeled wild-type or mutant PCGEM1; after pull-down, microRNAs were extracted, and the amount of miR-182 was assessed by qRT-PCR. **d** HeLa and SiHa cells were cotransfected with miR-182 mimics and luciferase reporters containing wild-type or mutant PCGEM1. After 48 h, luciferase activity was measured and presented as the relative ratio of firefly luciferase activity to renilla luciferase activity. **e** Anti-AGO2 RIP was performed in HeLa and SiHa cells transfected with miR-NC or miR-182, followed by qRT-PCR to detect PCGEM1 associated with AGO2. **f** SiHa cells were transfected with wild-type or mutant PCGEM1. After 48 h, the miR-182 expression was assessed by qRT-PCR. **g** HeLa cells were transfected with PCGEM1 siRNAs. After 48 h, the miR-182 expression was assessed by qRT-PCR. **h** The expression of miR-182 in 68 pairs of CC and normal cervical tissues was determined by qRT-PCR. **i** Expression levels of PCGEM1 and miR-182 were subjected to Pearson correlation analysis. *p < 0.05, **p < 0.01, ***p < 0.001

We then investigated the mechanism of the effect of PCGEM1 on CC cells. Because the functions of lncRNAs depend on their subcellular distributio, we first examined the cellular localization of lncRNA PCGEM1. Using cytoplasmic and nuclear RNA fraction assay from CC cells, we observed that PCGEM1 was expressed in relative abundance in the cytoplasm (Fig. 4a), indicating that PCGEM1 may regulate gene transcription through association with miRNAs in a post-transcriptional manner. The online software miRcode (http://www.mircode.org/) was utilized to screen miRNAs that have complementary base paring with PCGEM1 (Additional file 1: Fig. S1). miR-182, miR-216a, miR-124a, miR-124b and miR-506 was identified to associate with PCGEM1. To validate this result, we performed MS2-binding sequences-MS2-binding protein-based RIP assay (MS2-RIP) and found that only miR-182 could be significantly pulled down by PCGEM1 (Fig. 4b). The RNA pull-down assay further demonstrated a direct interaction between PCGEM1 and miR-182, while PCGEM1 with mutations in miR-182 targeting site (PCGEM1-mut) could not associate with miR-182 (Fig. 4c). For further confirmation, we constructed luciferase reporters containing wild-type (WT) or mutated miR-182 binding sites (PCGEM1-mut). The luciferase activity of cells transfected with PCGEM1 WT plasmid was significantly decreased by miR-182 mimics, while there was no difference in cells transfected with empty vector or PCGEM1-mut plasmid (Fig. 4d). To assess whether PCGEM1 was regulated by miR-182 in an AGO2-dependent manner, we conducted anti-AGO2 RIP in CC cells transiently overexpressing miR-182. Endogenous PCGEM1 pull-down by AGO2 was specifically enriched in miR-182-transfected cells (Fig. 4e). Overexpression of wild-type PCGEM1 instead of the mutant PCGEM1, significantly reduced the levels of miR-182 in SiHa cells (Fig. 4f). Conversely, miR-182 expression was increased by depletion of endogenous PCGEM1 expression in HeLa cells (Fig. 4g). The expression of miR-182 was downregulated in CC tissues compared with normal tissues (Fig. 4h). Additionally, we found a dramatically negative correlation between PCGEM1 and miR-182 in CC tissues in CC tissues (Fig. 4i). Taken together, these results suggest that miR-182 is a bona fide PCGEM1-targeting microRNA.

### PCGEM1 exerts oncogenic effects through miR-182

**Fig. 5:**
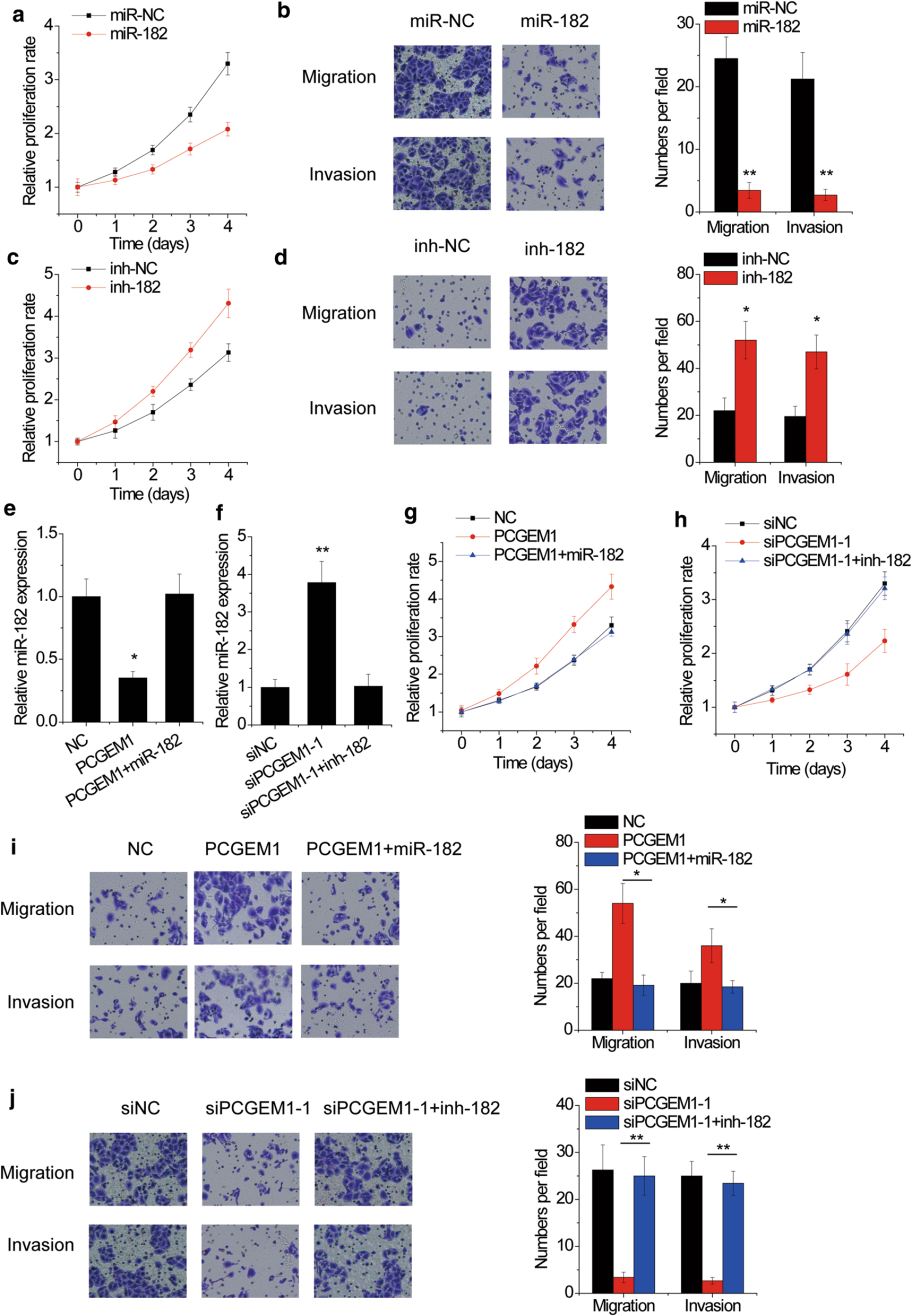
PCGEM1 exerts oncogenic effects through miR-182. **a** The proliferation of HeLa cells transfected with miR-NC or miR-182 mimics was assessed by CCK-8 assay. **b** Transwell assay was performed in HeLa cells transfected with miR-NC or miR-182 mimics. **c** The proliferation of SiHa cells transfected with miR-NC or miR-182 inhibitor was assessed by CCK-8 assay. **d** Transwell assay was performed in SiHa cells transfected with miR-NC or miR-182 inhibitor. **e** The miR-182 mimics were transfected into PCGEM1 overexpressing SiHa cells, and the miR-182 expression was examined by qRT-PCR. **f** The miR-182 inhibitors were transfected into PCGEM1 silencing HeLa cells, and the miR-182 expression was examined by qRT-PCR. **g** miR-182 abolished the promotion of cell proliferation mediated by PCGEM1 overexpression in SiHa cells. **h** miR-182 inhibitors abolished the suppression of cell proliferation mediated by PCGEM1 siRNAs in HeLa cells. **i** miR-182 abolished the promotion of cell migration and invasion mediated by PCGEM1 overexpression in SiHa cells. **j** miR-182 inhibitors abolished the suppression of cell migration and invasion mediated by PCGEM1 siRNAs in HeLa cells. *p < 0.05, **p < 0.01

To investigate the miR-182 effect on CC, we detected CC cell proliferation, migration and invasion after miR-182 overexpression or inhibition. The cell proliferation, migration and invasion was inhibited by miR-182 overexpression in HeLa cells (Fig. 5a, b), while miR-182 knockdown showed the opposite effects in SiHa cells (Fig. 5c, d). To determine whether the oncogenic effects of PCGEM1 were mediated by miR-182, CC cells with PCGEM1 alteration were transfected miR-182 mimics or inhibitor (Fig. 5e, f). It was shown that miR-182 mimics abolished the promotion of proliferation, migration and invasion mediated by PCGEM1 upregulation (Fig. 5g, i). In contrast, transfection with miR-182 inhibitor rescued the inhibitory effect of PCGEM1 siRNAs on cell proliferation, migration and invasion (Fig. 5h, j). Together, these data confirmed that miR-182 mediates the oncogenic effects of PCGEM1 in CC cells.

### FBXW11 is a target of PCGEM1 via miR-182

**Fig. 6:**
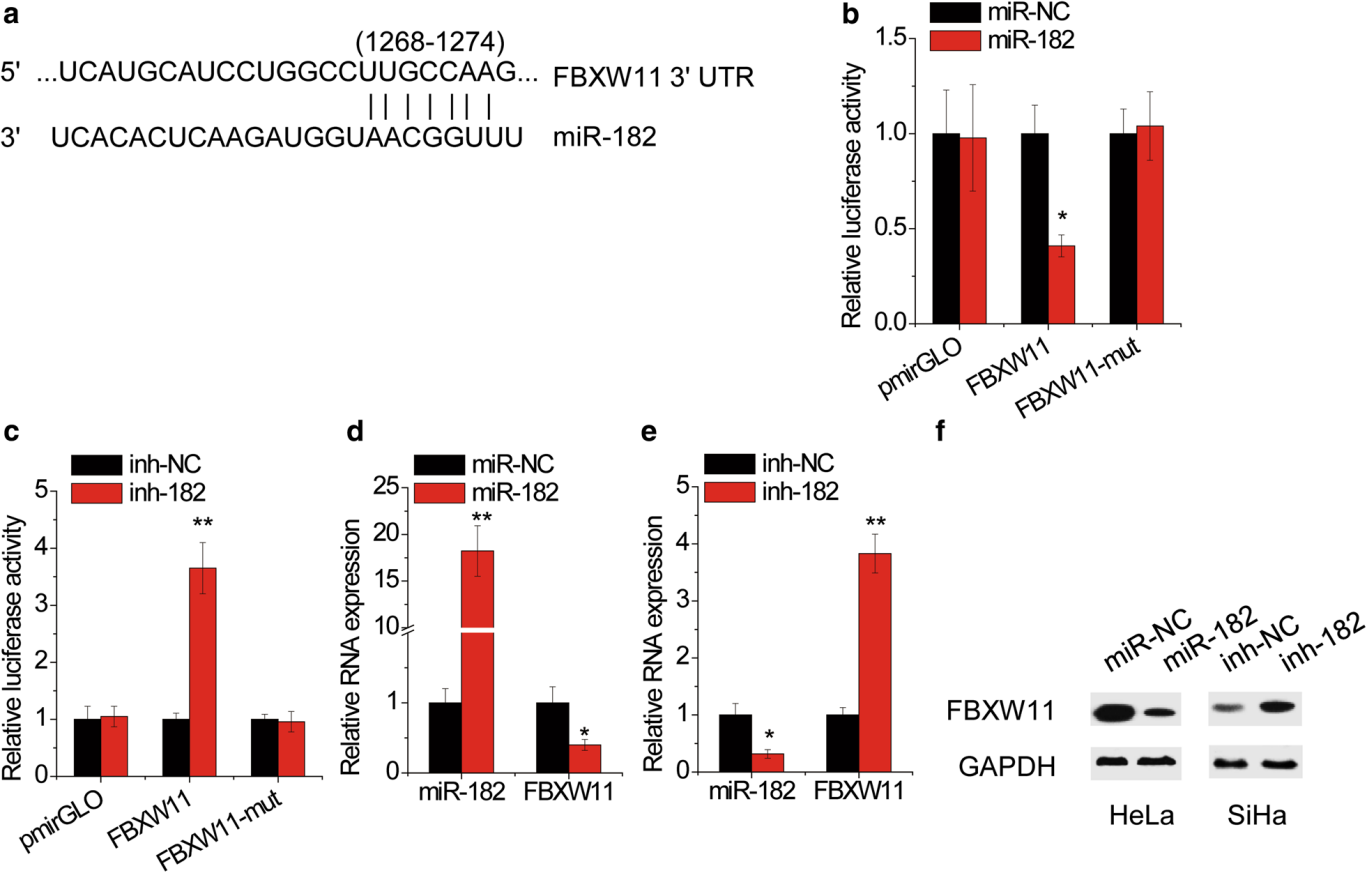
miR-182 targets FBXW11. **a** Prediction for miR-182-binding elements on FBXW11 3′UTR. **b** HeLa cells were cotransfected with miR-182 mimics and luciferase reporters containing wild-type or mutant FBXW11 3′ UTR. After 48 h, luciferase activity was measured and presented as the relative ratio of firefly luciferase activity to renilla luciferase activity. **c** SiHa cells were cotransfected with miR-182 inhibitors and luciferase reporters containing wild-type or mutant FBXW11 3′UTR. After 48 h, luciferase activity was measured and presented as the relative ratio of firefly luciferase activity to renilla luciferase activity. **d** HeLa cells were transfected with miR-NC or miR-182 mimics. 48 h later, the FBXW11 mRNA levels were detected by qRT-PCR. **e** SiHa cells were transfected with miR-NC or miR-182 inhibitors. 48 h later, the FBXW11 mRNA levels were detected by qRT-PCR. **f** HeLa and SiHa cells were transfected with miR-182 mimics and inhibitors, respectively. 48 h later, the FBXW11 protein levels were detected by western blot. *p < 0.05, **p < 0.01

To explore the target of PCGEM1-miR-182, informatics tools of three miRNA target-prediction programs (TargetScan, miRDB and miRcode) were used to search for the candidate targets and found oncogene FBXW11 3′UTR contains the binding sits of miR-182 (Fig. 6a). We performed luciferase reporter assays to confirm that the luciferase activity of wild-type FBXW11 3′UTR instead of its mutant was significantly inhibited by transfection of miR-182 mimics (Fig. 6b), but increased by miR-182 inhibitor (Fig. 6c). Furthermore, the mRNA and protein expression of FBXW11 was significantly reduced by miR-182 overexpression in HeLa cells (Fig. 6d, f). Conversely, miR-182 knockdown increased the expression of FBXW11 in SiHa cells (Fig. 6e, f).

**Fig. 7:**
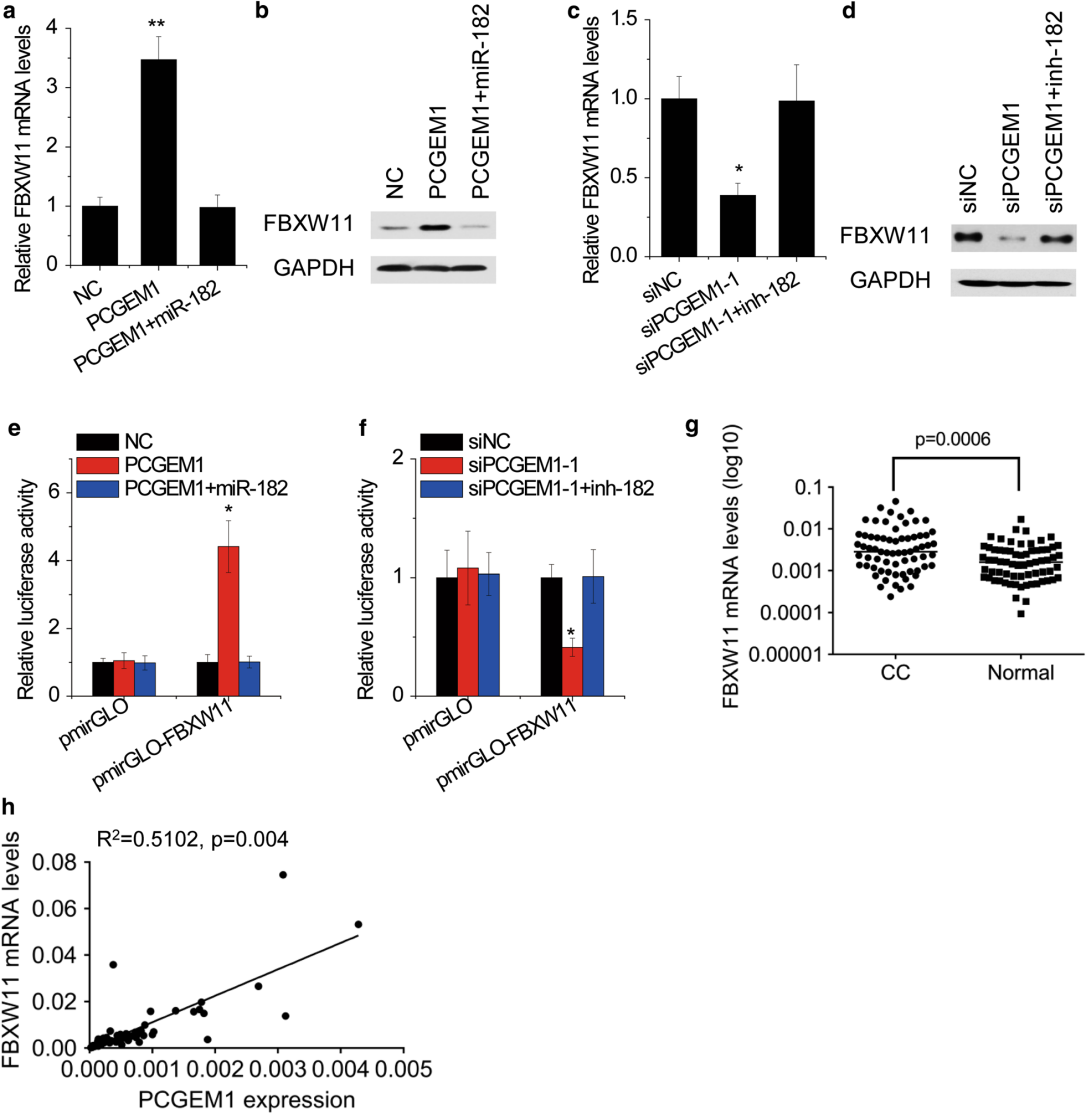
FBXW11 is a target of PCGEM1 via miR-182. **a** SiHa cells were cotransfected with PCGMEM1 and miR-182 mimics. After 48 h, the FBXW11 mRNA levels were determined by qRT-PCR. **b** SiHa cells were cotransfected with PCGMEM1 and miR-182 mimics. After 48 h, the FBXW11 protein levels were determined by western blot. **c** HeLa cells were cotransfected with PCGMEM1 siRNAs and miR-182 inhibitors. After 48 h, the FBXW11 mRNA levels were determined by qRT-PCR. **d** HeLa cells were cotransfected with PCGMEM1 siRNAs and miR-182 inhibitors. After 48 h, the FBXW11 protein levels were determined by western blot. **e** Luciferase activity in SiHa cells cotransfected with PCGEM1 and miR-182 and luciferase reporters containing FBXW11 3′UTR. **f** Luciferase activity in HeLa cells cotransfected with PCGEM1 siRNAs and miR-182 inhibitors and luciferase reporters containing FBXW11 3′UTR. **g** The expression of FBXW11 mRNA in 68 pairs of CC and normal cervical tissues was determined by qRT-PCR. **h** Expression levels of PCGEM1 and FBXW11 mRNA were subjected to Pearson correlation analysis. *p < 0.05, **p < 0.01

Next, we investigated whether PCGEM1 could regulate the expression of FBXW11. Our data showed that overexpression of PCGEM1 upregulated FBXW11 mRNA and protein expression in SiHa cells, which was attenuated by miR-182 overexpression (Fig. 7a, b). Conversely, FBXW11 expression was suppressed by PCGEM1 knockdown, which was rescued by inhibition of miR-182 (Fig. 7c, d). To ascertain whether this observed effect depends on regulation of the FBXW11 3′UTR, luciferase reporters plasmid containing FBXW11 3′UTR (pmirGLO-FBXW11) was constructed. pmirGLO-FBXW11, or the control reporter was transfected into CC cells with PCGEM1 alteration. Ectopic expression of PCGEM1 increased the luciferase activity of pmirGLO-FBXW11 in SiHa cells. miR-182 abolished this upregulation (Fig. 7e). Reciprocally, silence of PCGEM1 reduced the luciferase activity of pmirGLO-FBXW11, which was rescued by inhibition of miR-182 (Fig. 7f). The pathologic correlation of PCGEM1 and FBXW11 was then explored. FBXW11 was overexpressed in CC tissues compared with normal tissues (Fig. 7g). Notably, an obvious positive correlation between the levels of PCGEM1 and FBXW11 mRNA was observed in CC tissues (Fig. 7h). Thus, we concluded that FBXW11 was a direct target of miR-182 and positively regulated by PCGEM1 in CC cells.

### PCGEM1 activates NF-κB and β-catenin/TCF signaling pathways

**Fig. 8:**
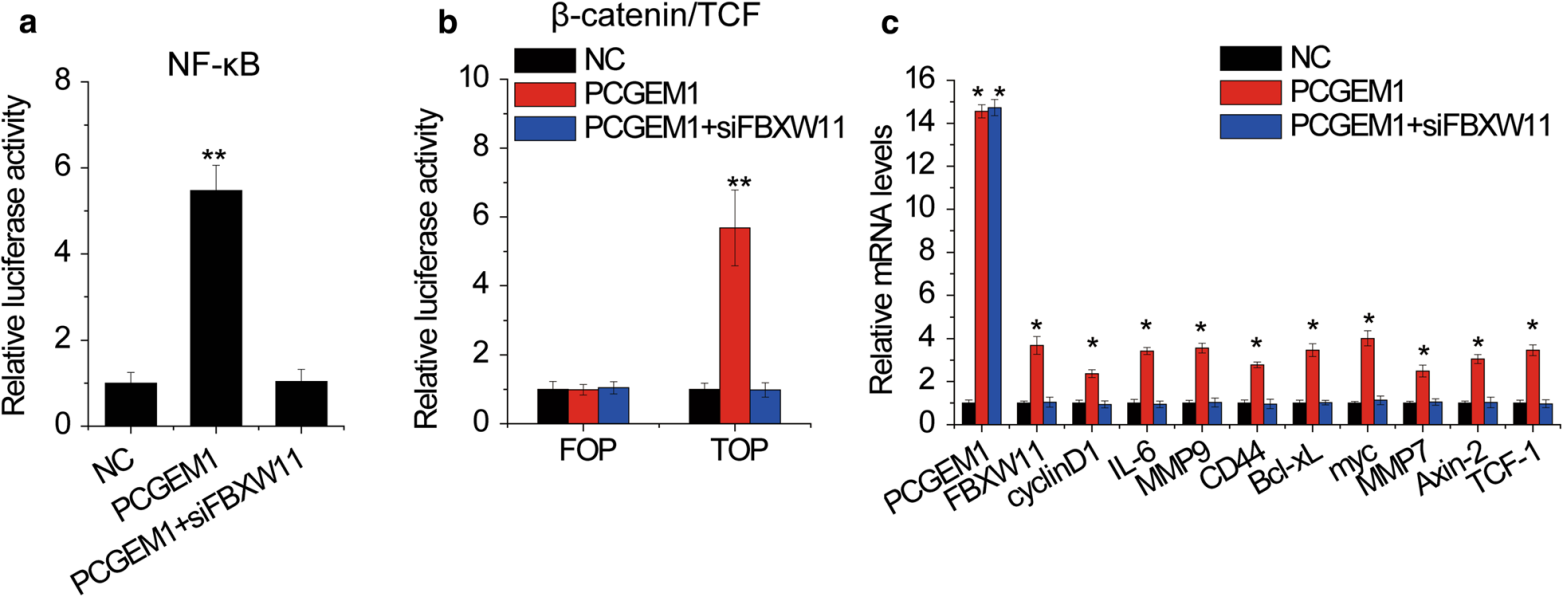
PCGEM1 activates NF-κB and β-catenin/TCF signaling pathways. **a** The dual luciferase method was used to analyze the activation of the NF-κB pathway in SiHa cells cotransfected with PCGEM1 and FBXW11 siRNAs. **b** The dual luciferase method was used to analyze the activation of the β-catenin/TCF signaling pathway in SiHa cells cotransfected with PCGEM1 and FBXW11 siRNAs, and a mutant TCF transcription factor (FOP) was used as a functional control. **c** The downstream gene of NF-κB and β-catenin/TCF signaling pathway was examined by qRT-PCR in in SiHa cells cotransfected with PCGEM1 and FBXW11 siRNAs. *p < 0.05, **p < 0.01

NF-κB and β-catenin/TCF play important roles in CC progression [23, 24]. Since FBXW11 could activate both the NF-κB and β-catenin/TCF signaling pathways [25], we suspected that lncRNA PCGEM1 may regulate these two signaling pathways through FBXW11. For confirmation, a dual luciferase reporter system was used and the results showed that both the NF-κB and β-catenin/TCF signaling pathways were enhanced by PCGEM1 overexpression, while knockdown of FBXW11 abolished this upregulation (Fig. 8a, b). Furthermore, we detected whether PCGEM1 affected the expression of downstream gene of NF-κB and β-catenin/TCF signaling pathways by qRT-PCR. NF-κB-regulated (including cyclinD1, IL-6, MMP9, CD44, Bcl-xL) and β-catenin/TCF-regulated (including myc, MMP7, Axin-2, CD44, cyclinD1, TCF-1) genes were markedly upregulated by PCGEM1. This upregulation was attenuated by silence of FBXW11 (Fig. 8c). The above data suggested that PCGEM1 promoted the activation of NF-κB and β-catenin/TCF signaling pathways via regulating of FBXW11.

## Discussion

Herein, we first demonstrated a tumor-promotive function of lncRNA PCGEM1 in CC cells. PCGEM1 was dramatically upregulated in both CC tissues and cell lines. High levels of PCGEM1 expression were associated with advanced FIGO stage, lymph node, distant metastasis, and worse overall survival of patients with CC. The similar prognostic value of PCGEM1 was also found in gastric cancer, ovarian cancer and prostate cancer [18, 20, 26]. Overall, our findings provided first clinical evidence that PCGEM1 may be used as a novel prognostic biomarker for patients with CC.

Recently, emerging evidence provided a novel regulatory mechanism between microRNA and lncRNA. lncRNAs associate with microRNAs, acting as ceRNAs by competitively binding common microRNAs [27]. Specifically, many miRNAs involved in CC progression could be regulated by lncRNAs. For instance, lncRNA DLG1-AS1 promotes cell proliferation by competitively binding with miR-107 and upregulating ZHX1 expression in CC [28]. LncRNA C5orf66-AS1, as a ceRNA, regulated the effect of RING1 on the proliferation, apoptosis and cell cycle of CC cells through sponging miR-637 [29]. In the present study, our group used bioinformatics assays and found that PCGEM1 contained binding sites for miR-182. The results of MS2-RIP, RNA pull-down and luciferase assays revealed a direct interaction between PCGEM1 and miR-182. Our findings also showed that miR-182 expression was suppressed by PCGEM1 and negatively correlated with PCGEM1 expression in CC tissues. Moreover, miR-182 is responsible for PCGEM1-miediated CC cell proliferation, migration and invasion. These data suggested that PCGEM1 exerted oncogenic effect on CC progression by negatively regulating miR-182 expression.

FBXW11 belongs to the FBXW subfamily of the F-box protein family, which is crucial for embryonic development and plays pivotal roles in various signaling pathways by regulating the ubiquitination of phosphorylated substrates [30]. Recent studies demonstrated that deregulation of FBXW11 contributes to carcinogenesis. For example, FBXW11 plays an important role in controlling the IκB-dependent apoptotic pathway in human melanoma and colorectal cancer [31]. FBXW11 facilitates skin tumor progression by activating the NF-κB signaling pathway [32]. Moreover, FBXW11 promotes the activation of the NF-κB and β-catenin/TCF signaling pathways, inducing cell cycle progression and tumor formation in lymphocytic leukemia [25]. However, the role of FBXW11 in CC progression remains elusive. Here, we demonstrated a dramatically increase of FBXW11 expression in CC tissues. Consistent with previous research [33, 34], our results identified PCGEM1 as a direct target of miR-182. Interestingly, lncRNA PCGEM1 could upregulate FBXW11 through acting as a ceRNA for miR-182. We also found that PCGEM1-mediated upregulation of FBXW11 activated NF-κB and β-catenin/TCF signaling. Overall, our results indicated that PCGEM1 regulated FBXW11 in the carcinogenesis of CC by acting as a molecular sponge to modulate miR-182.

## Conclusion

**Fig. 9:**
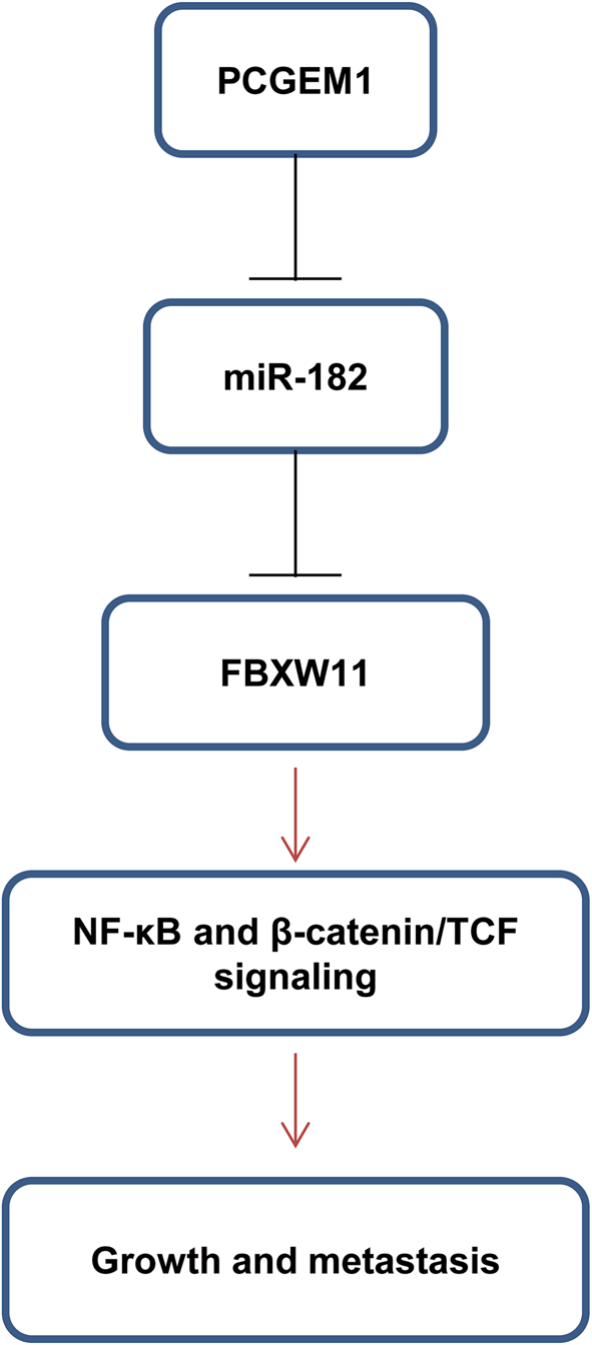
The schematic representation of PCGEM1-miR-182-FBXW11 axis in CC

Our study provides the first evidence that PCGEM1 promotes CC cell proliferation, migration, and invasion, as well as suppresses CC cell apoptosis and cell cycle arrest by targeting miR-182/FBXW11. Additionally, PCGEM1/miR-182/FBXW11 axis facilitates the activation of NF-κB and β-catenin/TCF signaling pathways (Fig. 9). These findings provide a new mechanism of CC progression and a promising prognostic biomarker of CC.

## Supplementary information

**Additional file 1: Figure S1.** The online software miRcode (http://www.mircode.org/) was utilized to screen miRNAs that have complementary base paring with PCGEM1.

## Data Availability

The datasets used during this research are available from the corresponding author upon reasonable request.

## Abbreviations

CC: cervical cancer
lncRNAs: long noncoding RNAs
PCGEM1: prostate cancer gene expression marker 1
ceRNA: competing endogenous RNA
EMT: epithelial–mesenchymal transition
FBXW11: F-box and WD repeat domain containing 11
DMEM: Dulbecco’s Modified Eagle Medium
qRT-PCR: quantitative real-time PCR
CCK-8: cell counting kit-8 reagent
RIP: RNA immunoprecipitation
MS2-RIP: MS2-binding sequences-MS2-binding protein-based RIP assay
FIGO: International Federation of Gynecology and Obstetrics
