# Supplementary figures and images for: The long noncoding RNA PCGEM1 promotes cell proliferation, migration and invasion via targeting the miR-182/FBXW11 axis in cervical cancer
Source: Cancer Cell Int. 2019 Nov 20;19:304. doi: 10.1186/s12935-019-1030-8 (PMC6865000; doi:10.1186/s12935-019-1030-8)

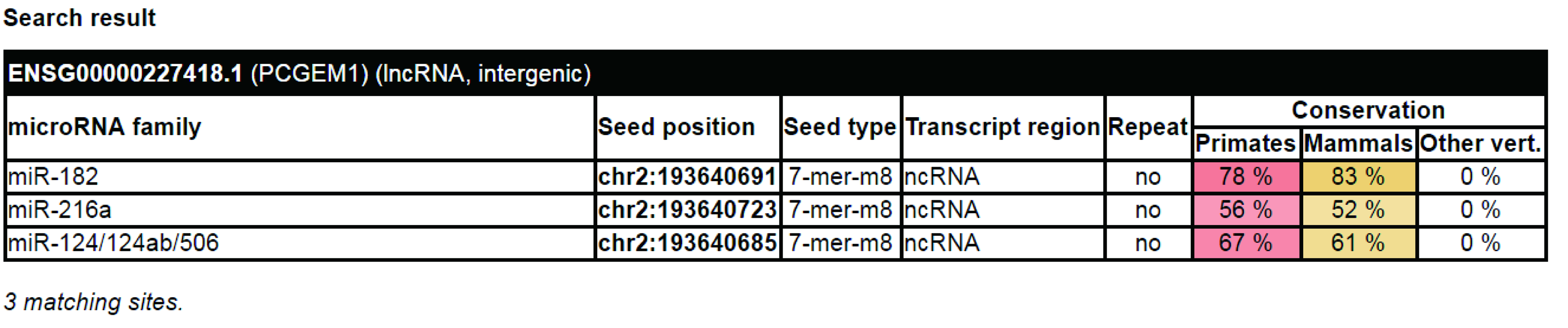

Supplement: Supplementary file 1 — Additional file 1: Figure S1. The online software miRcode (http://www.mircode.org/) was utilized to screen miRNAs that have complementary base paring with PCGEM1. [file 12935_2019_1030_MOESM1_ESM.tif]
